# Aboriginal Consumption of Estuarine Food Resources and Potential Implications for Health through Trace Metal Exposure; A Study in *Gumbaynggirr Country*, Australia

**DOI:** 10.1371/journal.pone.0130689

**Published:** 2015-06-22

**Authors:** Shaina Russell, Caroline A. Sullivan, Amanda J. Reichelt-Brushett

**Affiliations:** School of Environment, Science and Engineering and Marine Ecology Research Centre Southern Cross University, Lismore, Australia; Phillip Island Nature Parks, AUSTRALIA

## Abstract

Fishing and resource use continues to be an essential aspect of life for many Aboriginal communities throughout Australia. It is important for dietary sustenance, and also retains deep social, cultural and economic significance, playing a fundamental role in maintaining group cohesion, transferring cultural knowledge and affirming Indigenous identities. We surveyed approximately 20% of the *Gumbaynggirr* Aboriginal community of Nambucca Heads, New South Wales, Australia. This paper explores *Gumbaynggirr Connection to Country* and engagement in cultural practice. It quantifies fishing efforts and consumption of seafood within the community. We found 95% of the sample group fish, with the highest rate of fishing being 2-3 times a week (27%). Furthermore, 98% of participants eat seafood weekly or more frequently, up to more than once a day (24%). Survey results revealed that *Myxus elongatus* (Sand mullet) and naturally recruited *Saccostrea glomerata* (Sydney rock oysters) continue to be important wild resources to the *Gumbaynggirr* community. Trace metals were measured in *M*. *elongatus* and *S*. *glomerata* samples collected by community participants in this study. Maximum levels prescribed in the Australia New Zealand Food Standards Code were not exceeded in the edible tissue for either species, however both species exceeded the generally expected levels for zinc and copper and *S*. *glomerata* samples exceeded the generally expected level for selenium. Furthermore the average dietary exposure to trace metals from consuming seafood was calculated for the surveyed population. Trace metal intake was then compared to the provisional tolerable weekly intake prescribed by the Joint Expert Committee on Food Additives. This process revealed that copper and selenium intake were both within the provisional tolerable weekly intake, while there is no guideline for zinc. Furthermore, participants relying heavily on wild resources from the Nambucca River estuary may exceed the provisional tolerable weekly intake for cadmium. This suggests the need for further investigation of this issue to minimize any possible health risk.

## Introduction

Throughout history and prehistory there is evidence that Aboriginal people have been associated with aquatic ecosystems including rivers, lakes and the sea [1, 2]. Aquatic resources have been depended upon for subsistence, cultural, social, customary and economic reasons [1–4]. Coastal land and sea continue to be important societal and cultural environments for Aboriginal people today [3]. These environments have shaped the identity of populations and represent important cultural heritage [5]. Following European colonization, western land-use activities reduced hunting opportunities and availability of resources; consequently Aboriginal people were forced to rely increasingly on aquatic resources from rivers and oceans [6].

Cultural fishing and gathering of food resources in Aboriginal communities provides access to a reliable source of protein, but also underpins holistic health of individuals and the community; through maintaining family relations (kinship), affirming Indigenous identities, facilitating the continuity of cultural transmission and supporting the growth and transfer of traditional ecological knowledge [2, 7, 8, 9]. There is also considerable emphasis placed on the educational role associated with fishing, hunting and gathering. These acts are at the core of teaching young people about *Country* and their special responsibilities under customary *Lore* [6, 10]. Traditionally in Aboriginal culture, fishers retained an innate responsibility to provide for their family and the wider community [9, 11]. These cultural expectations and traditions are still exercised within Aboriginal communities today [2, 12, 13] and are largely dependent on local history, tenure and legislation [14]. Australian Indigenous culturally specific terminology has been presented in italics to ensure intention of words maintain integrity when received by a wider international community. i.e. *Gumbaynggirr*, *Connection to Country*, *Traditional Owner*, *Country* and *Lore*.

In recent years, there has been recognition of Indigenous sea rights by some western nations; Canada and New Zealand are among those who have initiated acknowledgement [15–17]. Aboriginal fisheries in Australia are receiving state as well as overarching nationwide recognition due to the persistent Indigenous advocacy for fishing rights over many years [9, 14]. These changes followed the Mabo court case of 1992 [18] and subsequent *Native title Act 1993* (Cth) [19]. Under the Act, *Traditional Owners* have the right to take marine resources, including the harvest of turtles and dugongs [20]. Following recognition of Indigenous rights to and involvement in the marine environment, the term traditional ecological knowledge (TEK) began to gain recognition. Integration of TEK with science and management knowledge (SMK) is beginning to occur, providing a rich body of knowledge for problem solving and essentially enhancing the resilience of social ecological systems [21–24].

In this paper, we examine the relationship between the Nambucca River estuary, and the use of food resources by the *Gumbaynggirr* People of New South Wales. While there are many foods consumed by these people, we focus here particularly on naturally recruited *Saccostrea glomerata* (Sydney rock oysters) and *Myxus elongatus* (Sand mullet). Both species are highly regarded among the *Gumbaynggirr* community for cultural, customary, and sustenance purposes, in both a historic and contemporary context. However due to their feeding techniques, both species have a high capacity for bioaccumulation of contaminants [25–27]: Oysters filter large quantities of water [28] and the fish family, Mugilidae, to which *M*. *elongatus* belong are largely detritivores [25, 26]. Human consumption of these species may lead to undesirable health implications.

Trace metals persist in the environment and exist within aquatic ecosystems from natural and anthropogenic origins [28–31]. They are widespread and have a tendency to accumulate in the tissue of many aquatic animals [28, 32, 33, 34, 35]. Presence of trace metals in the Nambucca River estuary may be due to the influence of derelict mines, agriculture, cattle dips and mineralization in the catchment [35, 36]. Prior to the 1970s, horticulture and banana growing activities included the use of early generation chemical sprays and fertilizers. Many of these chemicals are now banned; however, they continue to persist in the environment. Some that have been used in the area include metal-based sprays of arsenic and lead, organochlorine pesticides including DDT, dieldrin, aldrin and organophosphate pesticides [36].

While some metals are essential for living organisms, aiding to stabilize protein structures, facilitate electron transfer and catalyze enzymatic reactions [31], even biologically essential metals can be harmful if levels exceed certain thresholds [31, 37]. The effects of trace metals on organisms can range from acute mortality, to chronic effects including reduced growth rate and reproduction [30]. In extreme cases, humans who have high levels of trace metals display neurological disorders, cancer, carcinogenic action, bone deterioration and immune system disorders and such responses are often dependent on the metal of interest [38, 39]. Mercury, arsenic, cadmium and lead are the metals of most concern from a human health perspective [31, 38, 40], and in many parts of the world, including Australia, guidelines have been introduced on the maximum permitted levels of these metals in seafood for human consumption [41]. The Joint Expert Committee on Food Additives has also generated guidelines outlining the provisional tolerable weekly intake for some metals [42–44] and the United States of America Environmental Protection Agency [45] has released a reference dose for some metals. While the health implications of trace elements are widely recognized, exposure continues to occur [31, 39, 40].

At present, 2.6 billion people Worldwide derive their main source of protein from the ocean, Coastal communities and islander societies in arid regions rely on marine sources for up to 90% of their protein intake [5]. As the World’s oceans are increasingly affected by anthropogenic activities, the above figures highlight the vulnerability of these communities [5]. Furthermore, some population sub-groups may be susceptible to increased health effects due to dietary habits exposing them to greater levels of contaminants than the rest of the population [45–49]. For example, in the United States of America, mercury contamination of localized fish stocks has created a disproportional threat to Native American populations who rely on particular fisheries for subsistence and fulfillment of ritual culture [50, 51]. Similarly, indigenous people in the Arctic rely heavily on marine mammals of high trophic status, in which some contaminants are biomagnified, exposing people to elevated levels of mercury and Persistent Organic Pollutants (POPs) [47, 48, 50]. Furthermore, Torres Strait Islanders have become increasing concerned about culturally significant marine food resources since high cadmium concentrations were discovered in the liver and kidney of dugong and turtle in 1996 [52]. A study conducted by Haswell-Elkins et al [52] found cadmium in two Torres Strait Islander communities were largely associated with age, being female and smoking and suggestive links with having diabetes, a higher body fat percentage and living in a community with higher dugongs and turtle catch rates [52].

In this respect, coastal Aboriginal peoples’, where aquatic resources are central to culture, custom and diet, may represent a population sub-group vulnerable to such risks [47, 48, 50, 53]. If such a risk was to be recognized, adoption of lower intake guidelines may decrease the use of these traditional foods. While this would result in lower levels of contaminant intake, substitution of the traditional diet with processed foods high in starch, fat and sugar may result in alternative health implications, including increased risk of diabetes and cardiovascular disease [27, 53, 54]. Furthermore, movement away from relying on cultural foods may facilitate the loss of cultural transmissions and cultural identity [50]. This suggests that there is an urgent need to identify and remediate possible sources of contamination rather than reduce traditional food consumption.

In this paper a multidisciplinary approach was undertaken, combining complimentary socioeconomic and contaminant assessments to determine threat to Indigenous health from consuming cultural food resources. A questionnaire was used to explore peoples’ *Connection to Country* and to gain an estimation of dietary intake of estuarine resources. Concurrently chemical analyzes were completed on samples of two widely consumed species, to determine concentrations of trace metals that may pose a threat to human health. Based on this information, the approximate quantity of contaminant ingested was calculated and compared to the Joint Expert Committee on Food Additives provisional tolerable weekly intake [42–44] and the reference dose prescribed by the United States of America Environmental Protection Agency [45].

## Methods

### Study site

The study took place in Southern *Gumbaynggirr Country*, Nambucca Heads, NSW, Australia, with the *Traditional Owners* of the region; the *Gumbaynggirr* People. According to the 2011 Census the population of Aboriginal people in Nambucca Heads was 269 [55]. However, due to low literacy levels, substance abuse, and disengagement from Government, these population estimates may not be truly representative of the community and the Aboriginal population of the area is likely to be much higher. The unemployment rate of the area is 18.3%, more than double that of the state average (7.2%) [55]. Because of the high Indigenous population coupled with high unemployment rate, it is likely that the number of people using the wild resources of the Nambucca River is a significant proportion of the Indigenous population.

### Socioeconomic assessment

A total of 60 respondents ranging in age from 18 to 65 years and above participated in the research by completing a 42-question survey after written informed consent was received. The survey and consent procedure was reviewed and approved through the Southern Cross University Human Research Ethics Committee: ethics approval number ECN-13-104. The aim was to survey a broad range of age groups with a gender balance to be representative of the whole community. On the basis of the most recent official numbers of the Aboriginal people in Nambucca Heads, the sample size represents approximately 20% of the Indigenous population [55]. Furthermore, participants reported household consumption quantities, which may be representative of the broader community. Prior informed consent was obtained on a community level prior to commencement of the research, and on an individual level prior to participation in the research [56].

The questionnaire gathered quantitative and qualitative data on *Gumbaynggirr* use of estuarine food resources and explored respondents’ *Connection to Country*. A “funnel technique” was used in the questionnaire [57]; questions became slightly more detailed and focused as the survey progressed. Most questions were closed ended, while some required responses to be scored on a scale between 1–7. For the purpose of these analyzes, just the responses scoring 1 (best) were analyzed. It is possible this may underestimate the overall importance to the wider community, but further analyzes are beyond the scope of this paper. Following these were open-ended questions designed to gain deeper personal insights on the topic. The questionnaire was pilot tested at a community meeting, prior to delivery to the community. The pilot aimed to test understandability and recommended amendments were made following the trial.

Questionnaires were administered on a face-to-face basis; this delivery mode was a way to moderate veracity of answers. Where possible, data triangulation was provided through discussion with key informants. Participants were invited to a short presentation on the project to provide feedback. The results were clearly explained and participants were then asked if these results could be taken as being a representation of the views of the community as a whole. Participants agreed with the results of the questionnaire. Qualitative data was categorized into groups with common themes [58]. Concepts, themes and quotes formed a rich body of qualitative data, which supported and enriched the quantitative findings. Quantitative data from the questionnaire was converted into percent male and female and displayed in graphs and tables.

### Contaminant assessment

Triplicate *S*. *glomerata* and *M*. *elongatus* from three sample sites for each species were obtained from Aboriginal fishers using their normal fishing and gathering methods in a public fishing area. No permission was required to access the fishing locations. Animal ethics approval was gained through Southern Cross University Animal Care and Ethics Committee: ethics approval number 13/14. Samples were caught on the 16/5/2013 and 12/6/2013 for *S*. *glomerata* and *M*. *elongatus* respectively. Fish were caught by Aboriginal fishers using their standard practices. No endangered or protected species were collected. For analyzes, *S*. *glomerata* were dissected into gills and body and *M*. *elongatus* were dissected into gills, liver and muscles (without skin).

*M*. *elongatus* and *S*. *glomerata* tissue samples were dried at 80°C for at least 48 hours, samples were stored in a desiccator during cooling and prior to obtaining a dry weight. Dried tissue samples were crushed and weighed into small acid cleaned beakers to determine the weight of material to be digested after potential loss from the crushing process. After addition of 70% analar grade nitric acid, the material was refluxed for 1.5 hours at 75–80°C and cooled overnight.

After digestion, samples were filtered through glass fibre filter papers. Each set of digests included 2 blanks and 2 standard reference materials (DORM-4). Trace metals were analyzed by inductively coupled plasma mass spectrometry (ICP-MS) [30]. This method generally resulted in good metal recovery from the certified reference material (DORM-4) for; arsenic (As), 88%; cadmium (Cd), 90%; chromium (Cr), 86%; copper (Cu), 102%; lead (Pb), 60%; selenium (Se), 71%; and zinc (Zn), 85%.

Average dietary exposure was calculated by multiplying dietary intake based on number of meals reported by participants of *M*. *elongatus* and *S*. *glomerata*, by the level of contaminant present in edible tissue samples [59].

## Results

The compilation of results showed that 95% of the sample group (n = 60) gathered and/or fished in the Nambucca River estuary, with only 5% who did not. The sample group had a gender balance, with 55% of participants being women.

### Frequency of fishing and seafood consumption

Table 1 shows the frequency of fishing recorded by respondents, there were similar fishing efforts by both men and women, with most participants fishing 2–3 times per week (27%). These results also highlight a strong reliance on seafood consumption by Indigenous residents of Nambucca Heads. Table 1 shows that 24% of respondents consume fish more than once a day, with a further 31% eating fish once a day, and only 2% never consume fish. When considering the wider population, beyond the respondents themselves, the data indicates that on average, 96% of people in the participants’ households also consume food resources from the Nambucca River estuary. Consumption frequency of specific species is illustrated in Table 1.

**Table 1:** Fishing frequency and consumption.

		Frequency (%)							
	n	> 1 x day	Every day	2–3 times a week	Weekly	Once a month	6 + times a year	Annually	Never
Fishing	57		7	23	18	23	17	7	5
Seafood consumption	59	24	31	27	17				2
*S*. *glomerata* consumption	59		10	27	22	14	19	3	5
*M*. *elongatus* consumption	59		7	31	14	29	12	3	5

Frequency of the *Gumbaynggirr* communities fishing and resource consumption from Nambucca River estuary.

### Seasonal use of resources

**Fig 1:**
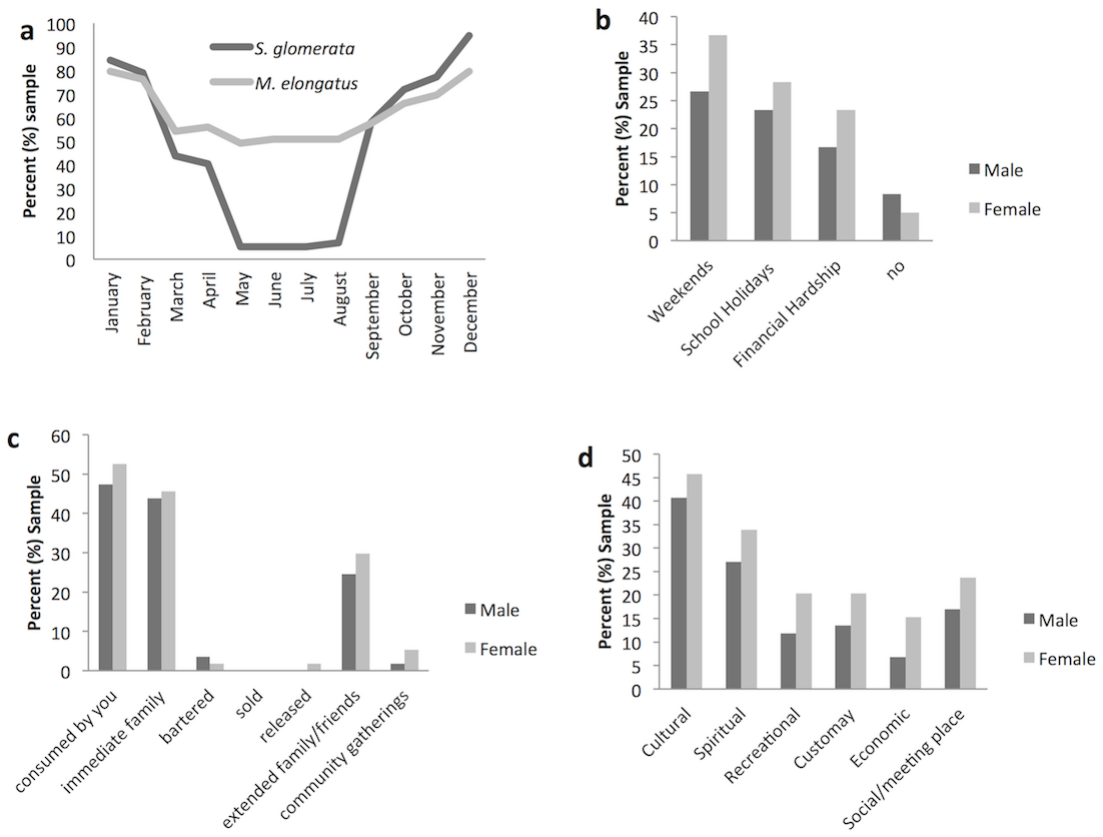
Seasonality, dependence and destination of catch, and significance of the Nambucca River estuary. (a) Seasonal use of *Saccostrea glomerata* (Sydney rock oyster) and *Myxus elongatus* (Sand mullet) (n = 57), (b) times of dependence on resources (n = 60), (c) destination of catch (n = 57), (d) significance of the Nambucca River estuary (n = 59) (n = number of respondents).

From the results it is clear that there is a strong seasonal influence on *S*. *glomerata* gathering, with 95% of participants harvesting in December and peak harvesting occurs from November to February. From May to August less than 10% of people surveyed collected oysters (Fig 1a). The seasonal division was not as evident for *M*. *elongatus*, where 51% of respondents said *M*. *elongatus* was consumed on a seasonal basis (Fig 1a).

### Seasonal dependence and consumption of catch

Fig 1a to 1d provide a summary of responses from a selection of questions, which indicate variations in timing of use, and beneficiaries of fishing effort. It was clear that preferred fishing and gathering activities were during weekends (63%) and school holidays (52%). It was also interesting to note that importantly, 40% of participants indicated that they experienced increased dependence on wild resources from the Nambucca River estuary during periods of financial hardship (Fig 1b). As shown in Fig 1c, out of the 95% of participants who fish in the Nambucca River, 100% consumed their own catch; 89% shared with family; 54% shared with extended family; 7% shared at community gatherings; and 5% bartered with their catch (Fig 1c). It was notable than no one sold their catch, but men were more likely than women to be involved in bartering.

### Significance of the Nambucca River estuary

Participants allocated an importance ranking for 7 aspects of the Nambucca River Estuary. Fig 1d shows that most participants revealed that the estuary was most important to them for cultural reasons (86%). Spiritual reasons were important for 61% of respondents, while 41% said the social aspect was the most important aspect of the estuary. A total of 34% identified customary reasons to be the most important. Recreational reasons were important for 32%, while only 22% said the estuary was most important for economic reasons (Fig 1d). Since participants were permitted to allocate highest significance to more than one attribute, the value of 100% was exceeded.

### *Gumbaynggirr* narrations

In the context of the cultural and spiritual values of fishing activities, some analyzes of the open-ended responses demonstrated the central nature of the estuary and its resources to these people. A selection of short quotations from the responses is provided in Table 2, which demonstrates five issues of particular relevance to the community. These few de-identified examples of responses collected in this research demonstrate the value of culturally relevant information and how it can support deeper understanding of different worldviews and approaches to fisheries resource management.

**Table 2:** Importance of the Nambucca River estuary.

Issue	Quotation from respondent
*Connection to Country*	*“The rivers*, *streams and lakes are sacred to Aboriginal people and are treated with respect like the land we live on*. *If the river doesn’t breathe then we don’t*, *it is a source of life as well as a source of food*.*”*
Values of fishing	*“Fishing and gathering is important for cultural aspects*, *it keeps the family together*. *Show the babies how to gather food for themselves*, *it’s not only about food gathering*, *but Lore*.*”*
Expression of identity	*“Fishing and gathering is important because it’s a part of our lives*, *it’s a part of our history*, *[and] it’s a part of our culture*. *We are the Sea people; all of our food comes from the sea and rivers*, *that’s why we lived on the river and the sea*. *We had everything in the water and everything in the forest*.*”*
Traditional ecological knowledge	*“All the travelling fish and how we know when they’re coming*. *All the grubs across the road*, *that’s the mullet and the little white butterflies*. *The fishing birds are out more*, *they’re more active*. *We watch for the wattle*, *that’s the blackfish*. *And when the ants are very active*, *that’s when it’s going to rain*. *Every little animals and plant*, *the Aboriginal people watch all that stuff*.*”*
Barriers to cultural fishing	*“They have to look at it in the Aboriginal way*, *we have a big family and it’s no good*, *the bag limits*, *because we have to feed the family*, *I like to feed the old people too*, *but that’s when you have to take a lot of kids*.*”*

Evidence of the importance of the Nambucca River estuary, from narrations gathered from survey participants.

### Trace metals and metalloids

From a human health perspective, the most important *M*. *elongatus* tissue analyzed was the muscle tissue. Trace metal concentrations for gills and liver have also been presented in Table 3, as they are an interesting indicator of ecosystem health. Furthermore metal analyzes of whole *S*. *glomerata* are reported here. Mean total arsenic concentrations in *M*. *elongatus* and *S*. *glomerata* were 4 mg/kg and 9 mg/kg respectively (Table 3). The Australia New Zealand Food Standards Code (FSANZ) [39] maximum level (ML) is reported as inorganic arsenic while this study presents the total arsenic concentration including both organic and inorganic forms. According to the literature inorganic arsenic constitutes approximately 20% of total arsenic in fish [60], while Spooner et al suggests that inorganic arsenic is never more than 1% in Australian marine animal tissues [61]. Further analytical investigation is necessary to determine arsenic speciation within biota.

**Table 3:** Trace metal concentrations in tissue samples of *Myxus elongatus* (sand mullet) and *Saccostrea glomerata* (Sydney rock oyster).

			Trace metal levels (mg/kg dry weight)				
		(Total)As	Zn	Pb	Cd	Cu	Se
*Myxus elongatus n = 9*							
Gills	Mean ± SD Range	7 ± 2 (5–11)	65 ± 24 (30–107)	2.33 ± 0.89 (1.29–3.98)	0.03 ± 0.01 (0.02–0.04)	8 ± 3 (6–10)	2.19 ± 0.47 (1.89–3.27)
Liver	Mean ± SD Range	15 ± 4 (6–21)	227 ± 85 (110–366)	0.55 ± 0.22 (0.23–0.99)	5.80 ± 3.00 (2.17–11.66)	128 ± 59 (45–229)	11.03 ± 4.51 (3.96–20.71)
Muscle	Mean ± SD Range	4 ± 1 (3–7)	39 ± 17 (24–74)	0.08 ± 0.12 (<0.01–0.39)	0.01 ± 0.01 (<0.01–0.02)	2 ± 1 (1–5)	0.97 ± 0.15 (0.83–1.26)
ML		2		0.5			
GEL (mean/95^th^ percentile)			5/15			0.5/2	
*Saccostrea glomerata n = 9*							
Gills	Mean ± SD Range	7 ± 4 (0.4–13)	4156 ± 3431 (144–12505)	0.3 ± 0.2 (0.2–0.8)	2.0 ± 1.7 (0.1–5.7)	207 ± 145 (10–497)	1.7 ± 0.7 (0.15–2.7)
Body	Mean ± SD	9 ± 5	2991 ± 2906	0.6 ± 0.3	2.6 ± 3.0	155 ± 126	2.9 ± 1.2
	Range	(5–18)	(858–9168)	(0.4–1.1)	(0.7–9.5)	(63–373)	(2.6–6.0)
Whole	Mean ± SD Range	9 ± 4 (1–16)	3935 ± 3170 (256–10620)	0.52 ± 0.23 (0.15–0.83)	2.67 ± 2.65 (0.25–8.47)	200 ± 134 (18–427)	2.79 ± 1.15 (0.66–5.09)
ML		1^†^		2	2 ^‡^		
GEL (mean/95^th^ percentile)			130/290			3/30	0.5

Mean, standard deviation (SD) and range of trace metal concentrations in tissue samples of *Myxus elongatus* (sand mullet) and *Saccostrea glomerata* (Sydney rock oyster) from the Nambucca River estuary compared with the maximum levels (MLs) and generally expected levels (GELs).

^†^ Guideline in inorganic arsenic

^‡^ Guideline for cadmium is for molluscs but excludes oysters

Mean lead concentration in *M*. *elongatus* and *S*. *glomerata* were 0.08 mg/kg and 0.52 mg/kg respectively (Table 3). Both were below the ML, however lead concentrations measured in both species should be treated with caution as they may be an underestimate of the actual lead concentration given the relatively low recovery (69%) of the certified reference material (DORM-4). Mean cadmium concentrations were 0.01 mg/kg and 2.67 mg/kg for *M*. *elongatus* and *S*. *glomerata* respectively. But ML values are not available to compare cadmium against the Food Standards Code (Table 3).

The mean concentration of zinc in *M*. *elongatus* and *S*. *glomerata* was 39 mg/kg and 3935 mg/kg respectively. Both values exceeded the respective generally expected level (GEL) of 5 mg/kg and 130 mg/kg for *M*. *elongatus* and *S*. *glomerata* (Table 3). *M*. *elongatus* and *S*. *glomerata* had mean copper concentrations of 2 mg/kg and 200 mg/kg respectively which exceeded the GEL of 0.5 mg/kg and 3 mg/kg for *M*. *elongatus* and *S*. *glomerata* respectively. Mean selenium concentration in *M*. *elongatus* and *S*. *glomerata* were 0.97 mg/kg and 2.79 mg/kg respectively, which exceeded the GEL of 0.5 mg/kg (Table 3).

## Discussion

By examining both the ecological health of food, and people’s usage of it, we gain a rich insight into human dependency on natural resources. In this study, this was achieved by detailed data gathering through engagement with the local community, while conducting contaminant analyzes of the tissues of two valued estuarine food species.

### Fishing frequency

In this study, 95% of respondents had engaged in wild aquatic resource collection in the estuary in the last 12 months. This highlights that fishing activities by the respondents are considerably higher than the state average of 60% (72% in remote communities) [62]; and is likely to be due to the coastal locality of the *Gumbaynggirr* community, and subsequent access to aquatic ecosystems. Henry and Lyle [63] found that in Northern Australia Indigenous persons (aged 5 years or older) present a regional fishing participation of 91.5% of the surveyed population, similar to the results of this study (95%). The frequency of fishing trips found in the *Gumbaynggirr* community (Table 1), are similar to findings elsewhere in New South Wales [9, 64].

### Total seafood consumption

Estimating total seafood consumption from the Nambucca River estuary was essential in understanding reliance on estuarine resources by the *Gumbaynggirr* community. It was also important in reference to the potential health implications imposed by consumption of fish and shellfish from the Nambucca River estuary. The results showed a very high reliance on wild resources by participants to supplement their diet (Table 1). This has also been confirmed by other studies in Australia [9, 63, 65]. To gain further insight into the community beyond the sample group, responses were sought on broader household consumption. The results revealed that 96% of respondents’ household members eat seafood from the Nambucca River estuary, with an average of 3.9 people per household (reported by participants). This suggests at least 234 people, representing 87% of the local Indigenous population living in Nambucca Heads, are consuming estuarine resources from the local river. This is also reflected in the data set as those involved in the research, shared most fish catch with immediate family (89%).

### Key species and seasonality

Indigenous knowledge and seasonal indicators can still be seen to inform resource collection and use today [22, 66]. For example, most participants collect *S*. *glomerata* during the summer months (95%) when they are in season and said to be “fat.” Less seasonal variation was evident for *M*. *elongatus* with 51% of respondents fishing for them seasonally. Similarly, the Bardi Aboriginal People of One Arm Point, Western Australia assess relative fatness of species in their environment including fish, turtles and shellfish; procuring species only when they are considered to be at the fattest phase: during specific seasons, at specific physiological life stages or through on-site evaluation [56].

### Times of dependence on resources

Fishing and gathering at times when children are present (weekends (63%) and holidays (52%)) was a common activity of the *Gumbaynggirr* community. Most respondents in this study believed that their children already did or would have a strong reliance on the food resources from the Nambucca River estuary (90%). Schnierer [9] also found that children regularly accompanied adults when fishing. The combined results highlight the educational role associated with this activity and further emphasizes the significance of family fishing to knowledge transmission, defining identity, maintaining kinship and *Connection to Country* [6, 10, 67].

The results showed that 40% of the *Gumbaynggirr* community relies on the Nambucca River estuary during periods of financial hardship. This supports the reports that social and economic hardship affects people’s activities in resource-dependent communities [13]. Considering the high rates of unemployment in Nambucca Heads (18.3%), the Nambucca River estuary as an open access resource is critical for the *Gumbaynggirr* community as it provides access to a free and semi-reliable source of protein [57].

### Cultural expectations

Through the *Lores* of kinship, an Aboriginal fisher’s responsibility is to provide for their immediate and extended family, particularly Elders [11]. Because of this cultural expectation, the final destination of respondents catch was of interest. Traditional practices of gathering for the community as well as bartering are still exercised to a small degree within the *Gumbaynggirr* community. Similar to findings associated with communities from the Tweed Heads Region about 350 kms north of Nambucca Heads [9, 64]; confirming that coastal resources are indeed still used for consumption, barter and trade [9, 11]. When *Gumbaynggirr* People speak of the final destination of their catch, they are passionate about resource management stating:

“*we only take what we need*.*”*

They also highlight aspects of kinship:

“*we look after the Elders*.”

Similar community sharing has been noted in other studies of traditional lifestyles. For example kinship in a Malay fishing community is underpinned by sharing: sharing of space, sharing of food and nurturing one another [68]. These strong societal values of simple yet deep family responsibilities and generational obligations have shaped and preserved cultural integrity that still remains today.

### Significance of the Nambucca River estuary

The cultural significance underpinning fishing practice has been observed widely [2, 8, 9, 13, 16, 17, 69, 70] and was also seen in this research. Fig 1d shows the cultural aspects associated with the Nambucca River estuary are the most important to respondents. Furthermore, 15% of respondents stated that each criterion were interrelated, and all come under the broad criteria of being important for cultural reasons.

The sociocultural aspect of fishing further emphasizes the importance of fisheries access for Indigenous communities, and such importance is gaining recognition from fisheries authorities. The *Fisheries Management Act 1994* [71], was amended in 2009, ‘to recognise the spiritual, social and customary significance to Aboriginal persons of fisheries resources and to protect, and promote the continuation of Aboriginal cultural fishing.’ This is a progressive step, however further legislative development may be required in order to safeguard the practices of Aboriginal cultural fishing in Australia. Further recognition should not only include acknowledgment of the sociocultural benefit and subsistence values of fishing (people using their own efforts for life support), but also invoke a responsibility in ensuring that contamination by upstream land use practices is minimized.

### Insights from the *Gumbaynggirr* narrations

As reflected in the narration text presented in Table 2, the *Gumbaynggirr* People have had a long connection to the Nambucca River estuary. In a recording of *Gumbaynggirr* Elder of the Nambucca area, the late Harry Buchanan speaks of caring for ancestors who have taken the forms of surrounding *Country*, trees, caves and the moon [72]. These words highlight the intrinsic link between spiritual, ceremonial, social, and economic life, and *Connection to Country*. There is a strong connection to ancestral engagement and *Gumbaynggirr dreaming* cosmology, which is embedded within the landscape, seascape and riverscapes [73].

#### Indigenous Identity and Traditional ecological knowledge

Cultural fishing remains important for spiritual and ceremonial purposes and the connection of coastal Aboriginal communities to both salt and freshwater underpins their identity [2, 5, 8]. The Southern *Gumbaynggirr* People are a saltwater rainforest clan, and their totem is the sea [74]. Participants of this study revealed the local environment and engagement in customary and cultural practice underpins their identity (Table 2).

Traditional ecological knowledge is grounded by a broad knowledge base of the behavior of complex ecological systems in a localized area [73]. Indigenous people have accumulated knowledge through continuity of resource use, and this knowledge has been transmitted from generation to generation [5, 73, 74]. These observations over time form a rich body of traditional ecological knowledge, which is gaining increasing recognition in Australia [21, 22, 24, 75, 76] and internationally [77–79] The *Gumbaynggirr* People have always been governed by customary traditions, sacred *Lore* and the seasons [74]. Tides, seasons and moon cycles would have historically influenced resource use and key environmental indicators are still seen to inform resource collection and use today. Following are some quotes offering examples of this:

*“Indicator species*, *every tree*, *every species tells you a story*. *You need to be watching and listening*.*”*

*“Uncle Eddy was a clever man*. *Uncle Eddy would see the butterflies off the coast and know the mullet were coming*.*”*

#### Institutional arrangements as barriers to cultural fishing

Fishing is an integral mechanism for passing on traditional knowledge, maintaining cultural connections and supporting family networks [80]. However the presence of contemporary fishing laws can make exercising and passing on customary and cultural practices difficult for Aboriginal people. This difficulty was identified in this study through comments from the *Gumbaynggirr* community, and also reflected by Schnierer [9] as a barrier to cultural fishing among the Tweed community. Participants identified that western laws, including bag limits, and gear restriction have inhibited cultural obligations, and subsequent capacity to fulfill cultural expectations. Furthermore the presence of contemporary laws has altered the way children engage with the river and resource gathering (Table 2). Although the *Fisheries Management Act 1994* [71] has been amended to include the Indigenous fishing sector, restrictions are still imposed which impact on cultural practices.

### Trace metals concentrations and implications for health

For the *Gumbaynggirr* population, the average dietary exposure to trace metals from consuming seafood was estimated (Table 4). Whole *S*. *glomerata* generally had relatively high concentrations of zinc, copper and selenium, and *M*. *elongatus* muscle tissue also contained elevated levels of zinc and copper. Lead did not exceed the maximum level in either species. Cadmium was elevated in *S*. *glomerata* and very low in *M*. *elongatus* (Table 3), however there is no guidelines set to assess such concentrations.

**Table 4:** Trace metal ingestion based on consumption of food resources.

% of surveyed population	Frequency of consumption	As (mg/kg) (Total)	Cd (mg/kg)	Cu (mg/kg)	Pb (mg/kg)	Se (mg/kg)
		Dietary exposure to metals according to quantity of seafood consumption				
*Myxus elongatus*						
7	Everyday	4.2	0.01	1.7	0.1	0.9
32	3 x per week	1.8	<0.01	0.7	0.04	0.4
14	1 x per week	0.6	<0.01	0.2	0.01	0.1
*Saccostrea glomerata*						
11	Everyday	4	1.2	89.6	1.2	0.2
29	3 x per week	1.7	0.5	64	0.1	0.5
14	1 x per week	0.6	0.2	12.8	0.03	0.2
Combined consumption (*Myxus elongatus* + *Saccostrea glomerata*)						
9	1 x each sp. x per day	8.2	1.2	91.3	0.3	2.2
30.5	3 x each sp. x per week	3.5	0.5	64.7	0.1	0.9
14	1 x each sp. x per week	1.2	0.2	13	0.05	0.3
PTWI (μg) based on average weight male (85.9kg) ^†^		1.3 (inorganic)	0.6	301	2.1	3^‡^
PTWI (μg) based on average weight female (71.1 kg) ^†^		1.1 (inorganic)	0.5	245	1.8	2.5^‡^
*Myxus elongatus*–based on serving size of 120g^§^						
*Saccostrea glomerata*–based on serving size of 64g^|^						

Percentage of sample population consuming a particular quantity of *Myxus elongatus* (sand mullet) and *Saccostrea glomerata* (Sydney rock oyster) from the Nambucca River estuary and subsequent quantity of contaminant ingested. Intake quantities are compared with the provisional tolerable weekly intake (PTWI).

^†^ Average weight from the Australian Bureau of Statistics (2013).

^‡^ Reference Dose (USAEPA, 1991).

^§^ Derived from the Australian Government (2005).

^|^ Derived from average weight of oysters (2g) multiplied by average number of oysters consumed in a sitting (32).

Participants relying heavily on food from the estuary may be exceeding their threshold for some metals. For example participants eating *S*. *glomerata* more than three times a week may be exceeding the provisional tolerable weekly intake (PTWI) for cadmium (Table 4). The setting of such values considers the bioavailability, uptake and urinary excretion of cadmium [81]. Table 4 should be used as a guide only, as it only takes into account the two species focused on in this study and uses estimated consumption quantities to derive implications for health. The 120-gram fish portion size is based on the Australian Government [82] dietary guidelines for Australians and anecdotal observations suggest that this may be an underestimate of Indigenous portion size. The average weight of *S*. *glomerata* [2g] is quite small, and as a constraint of this study, were collected at a time of lesser use when oysters are not considered to be ‘fat’ (Fig 1a), hence the results need to be considered within this limitation. Average number of *S*. *glomerata* consumed in a sitting was based on results from self-reporting by participants of their own consumption.

The questionnaire revealed that the proportion of total food from the estuary is higher than the consumption of *M*. *elongatus* and *S*. *glomerata* combined (Table 1). While these species are relied upon heavily during the warmer months, other species are also consumed throughout the year including, but not limited to, pipis, mudcrabs, cobra and a myriad of estuarine and sea fish species. Some participants indicated that they obtain over 90% of their food from the Nambucca River estuary, although this needs clarification in terms of species, it indicates a high reliance on the Nambucca River estuary. These findings warrant further investigation of trace metals and other contaminants in all food resources during seasonal peaks of consumption throughout the year from the Nambucca River estuary and coastal marine environment. This is particularly important in light of the relative consumption of seafood that may be different in portion sizes then the suggested dietary guidelines for Australians [82].

## Conclusion

In this study qualitative data collected from the Southern *Gumbaynggirr* community of Nambucca Heads provides insight into the importance of C*onnection to Country*, and significance of the Nambucca River estuary. This significance was reflected by high rate of fishing participation by the community, and a high reliance on food resources for sustenance from the Nambucca River estuary. Indigenous fishing efforts and rates of seafood consumption were quantified through the collection of quantitative data. Furthermore analyzes of fish and oyster tissue showed that dietary exposure to trace metals as determined through consumption rates was generally within the provisional tolerable weekly intake. However, it appears that some individuals relying considerably on wild resources from the estuary may be exceeding their provisional tolerable weekly intake for cadmium.

Considering the economic disadvantage and prevailing health issues faced by Aboriginal people today, their use of wild resources is likely to continue to be important. As a subpopulation with a strong reliance on aquatic resources, the *Gumbaynggirr* and other coastal people may be vulnerable to health risks, through the consumption of contaminated aquatic foods. The deep cultural and spiritual benefits for individuals and the community derived from engaging in customary practices has been emphasized throughout this research. Consequently care must be taken when considering contaminated resources, as restricting access may have negative effects on Indigenous cultural transmission, and potentially result in a different suite of health implications for the impacted community. Therefore effort to reduce upstream contamination rather than restrict access to the resource is necessary.

## Supporting Information

S1 File Trace metal concentrations in tissue samples of *Myxus elongatus* (sand mullet) and *Saccostrea glomerata* (Sydney rock oyster) from the Nambucca River estuary. (PDF)
