# Supplementary material for: Aboriginal Consumption of Estuarine Food Resources and Potential Implications for Health through Trace Metal Exposure; A Study in Gumbaynggirr Country, Australia
Source: PLoS One. 2015 Jun 22;10(6):e0130689. doi: 10.1371/journal.pone.0130689 (PMC4476661; doi:10.1371/journal.pone.0130689)
Supplement: S1 File — (PDF) [file pone.0130689.s001.pdf]

			Trace metal concentrations (mg/kg dry weight)					
M.elongatus			As	Zn	Pb	Cu	Cd	Se
Site 1	Replicate 1	Gills	7.02	69.80	2.43	6.94	0.04	2.39
		Liver	14.51	196.45	0.52	87.88	2.17	14.47
		Flesh	2.50	74.07	0.12	1.55	0.01	1.20
	Replicate 2	Gills	7.82	30.46	2.26	8.96	0.03	2.25
		Liver	17.58	159.25	0.67	99.76	7.23	14.94
		Flesh	6.38	66.50	-0.02	1.62	0.01	1.12
	Replicate 3	Gills	9.01	106.80	3.98	10.42	0.03	2.91
		Liver	17.71	282.78	0.73	163.88	7.97	20.71
		Flesh	5.34	34.00	0.01	1.58	0.01	1.26
Site 2	Replicate 1	Gills	6.04	75.25	2.07	10.22	0.02	1.86
		Liver	17.88	317.05	0.35	180.27	5.38	12.97
		Flesh	4.36	24.18	0.03	1.44	0.01	0.83
	Replicate 2	Gills	9.27	69.54	2.28	14.00	0.02	1.97
		Liver	17.45	341.84	0.66	229.36	7.92	12.85
		Flesh	4.48	31.87	0.03	1.10	0.00	1.15
	Replicate 3	Gills	10.75	79.01	2.98	13.07	0.03	1.99
		Liver	20.83	365.81	0.99	201.31	11.66	16.44
		Flesh	5.12	49.56	0.11	3.79	0.02	1.03
Site 3	Replicate 1	Gills	5.50	93.42	1.29	6.01	0.03	2.68
		Liver	6.06	110.36	0.23	45.01	2.59	3.96
		Flesh	3.03	36.26	0.01	0.91	0.00	0.93
	Replicate 2	Gills	8.17	40.37	2.10	7.45	0.04	2.55
		Liver	19.94	249.54	0.68	113.95	4.96	11.90
		Flesh	7.04	28.90	0.12	1.72	0.02	1.20
	Replicate 3	Gills	7.64	83.13	3.96	7.52	0.04	3.27
		Liver	16.38	250.21	0.66	154.81	8.17	11.14
		Flesh	4.96	42.36	0.39	4.78	0.02	0.97
S.glomerata								
Site 1	Replicate 1	Gills	11.87	6156.34	0.43	233.03	5.69	2.67
		Tissue	17.89	7056.19	0.50	299.18	9.48	5.97
	Replicate 2	Gills	13.46	5778.02	0.76	397.00	3.60	1.64
		Tissue	16.18	4810.64	0.76	327.53	6.92	2.95
	Replicate 3	Gills	10.60	3454.29	0.43	144.38	3.79	1.93
		Tissue	17.11	1247.16	0.36	69.21	3.04	3.81
Site 2	Replicate 1	Gills	7.43	3039.13	0.18	237.45	1.17	2.41
		Tissue	8.62	2033.64	1.09	129.04	1.31	2.67
	Replicate 2	Gills	6.70	3952.84	0.56	243.72	1.22	2.06
		Tissue	8.92	3445.45	0.66	202.14	1.61	3.32
	Replicate 3	Gills	6.57	2871.62	0.36	164.19	1.35	2.09
		Tissue	7.12	2063.26	0.52	154.77	2.43	3.26
Site 3	Replicate 1	Gills	0.36	144.40	0.04	10.09	0.11	0.15
		Tissue	5.85	858.09	0.75	62.98	1.03	3.41
	Replicate 2	Gills	4.05	3653.97	0.19	138.80	1.60	1.56
		Tissue	5.84	2175.73	0.30	83.82	0.74	2.56
	Replicate 3	Gills	4.30	12504.66	0.41	497.22	1.76	2.35
		Tissue	6.12	9167.86	1.00	372.84	1.83	3.39
